# Functional Health Literacy Among Chinese Populations and Associated Factors: Latent Class Analysis

**DOI:** 10.2196/43348

**Published:** 2023-04-28

**Authors:** Zhaogang Dong, Meng Ji, Yi Shan, Xiaofei Xu, Zhaoquan Xing

**Affiliations:** 1 Department of Clinical Laboratory Qilu Hospital of Shandong University Ji'nan China; 2 School of Languages and Cultures, The University of Sydney Sydney Australia; 3 School of Foreign Studies, Nantong University Nantong China; 4 Center for Reproductive Medicine Department of Obstetrics and Gynecology Qilu Hospital of Shandong University Ji'nan China; 5 Department of Urology Qilu Hospital of Shandong University Ji'nan China

**Keywords:** functional health literacy, associated factors, Chinese populations, latent class analysis

## Abstract

**Background:**

Poor functional health literacy has been found to be independently associated with poor self-assessed health, poor understanding of one’s health condition and its management, and higher use of health services. Given the importance of functional health literacy, it is necessary to assess the overall status of functional health literacy in the general public. However, the literature review shows that no studies of functional health literacy have been conducted among the Chinese population in China.

**Objective:**

This study aimed to classify Chinese populations into different functional health literacy clusters and ascertain significant factors closely associated with low functional health literacy to provide some implications for health education, medical research, and public health policy making.

**Methods:**

We hypothesized that the participants’ functional health literacy levels were associated with various demographic characteristics. Therefore, we designed a four-section questionnaire including the following information: (1) age, gender, and education; (2) self-assessed disease knowledge; (3) 3 validated health literacy assessment tools (ie, the All Aspects of Health Literacy Scale, the eHealth Literacy Scale, and the 6-item General Health Numeracy Test); and (4) health beliefs and self-confidence measured by the Multidimensional Health Locus of Control Scales Form B. Using randomized sampling, we recruited survey participants from Qilu Hospital affiliated to Shandong University, China. The questionnaire was administered via wenjuanxing. A returned questionnaire was valid only when all question items included were answered, according to our predefined validation criterion. All valid data were coded according to the predefined coding schemes of Likert scales with different point (score) ranges. Finally, we used latent class analysis to classify Chinese populations into clusters of different functional health literacy and identify significant factors closely associated with low functional health literacy.

**Results:**

All data in the 800 returned questionnaires proved valid according to the predefined validation criterion. Applying latent class analysis, we classified Chinese populations into low (n=292, 36.5%), moderate-to-adequate (n=286, 35.7%), and low-to-moderate (n=222, 27.8%) functional health literacy groups and identified five factors associated with low communicative health literacy: (1) male gender (aged 40-49 years), (2) lower educational attainment (below diploma), (3) age between 38 and 68 years, (4) lower self-efficacy, and (5) belief that staying healthy was a matter of luck.

**Conclusions:**

We classified Chinese populations into 3 functional health literacy groups and identified 5 factors associated with low functional health literacy. These associated factors can provide some implications for health education, medical research, and health policy making.

## Introduction

### Background

Health literacy refers to a range of skills and resources that are associated with the ability to process health-related information [1]. It is of major concern to health professionals and public health authorities [2]. It has been found that over one-fourth of the 31,129 participants in 85 studies had “inadequate” health literacy and another one-fifth had “marginal” health literacy [3]. Most studies used clinical cohorts that typically overrepresented socially disadvantaged groups, making it difficult to draw inferences about the overall status of health literacy in the general public [1]. No studies of functional health literacy have been conducted among the Chinese population.

It is increasingly recognized that interpersonal processes of care contribute to the overall quality of health care, in addition to technical processes of care [4-8]. Interpersonal processes comprise the social-psychological aspects of the clinical interaction, including patient-provider communication [9]. As a subset of health literacy skills involving interpersonal processes of care, functional health literacy has been defined as sufficient basic skills in reading and writing needed to function effectively in everyday situations [10]. Functional health literacy measures a patient’s ability to perform basic reading and numerical tasks essential for functioning in the health care context [11]. Limited functional health literacy is prevalent among patients with low educational attainment and among older patients and racial and ethnic minorities [11]. For example, as many as one-third of English-speaking Medicare patients have poor functional health literacy in a national managed care organization in the United States [12]. Similarly, other studies also reported high or even alarming figures of limited health literacy among particular populations in the United States: around 40 to 44 million adults [13], 41% of English-speaking adults 65 years or older [12], 53.9% of Spanish-speaking respondents [12], 81.3% of English-speaking patients 60 years or older [14], 82.6% of Spanish-speaking patients [14], among many others. As these figures show, poor functional health literacy is the rule rather than the exception in public sector settings [14]. To our knowledge, based on the literature review, no studies have investigated the functional health literacy status among Chinese populations in China. But we hypothesized that the prevalence of limited functional health literacy would also be high among Chinese populations in mainland China, given the high prevalence in the United States and informed by a very recent study that reported a high prevalence of critical health literacy among Chinese populations in China (221/588, 37.6%) [15].

Previous studies have investigated factors contributing to limited functional health literacy. Poor functional health literacy has been found to be independently associated with poor self-assessed health [12], poor understanding of one’s health condition and its management [16-18], higher use of health services [19,20], and so on. Given the importance of functional health literacy, it is necessary to ascertain factors closely associated with inadequate functional health literacy in the general public in China. However, no such studies have been conducted among the Chinese population.

### Objective

This study aimed to classify Chinese populations into different functional health literacy clusters and ascertain significant factors closely associated with low functional health literacy to provide some implications for health education, medical research, and public health policy making.

## Methods

### Questionnaire Design

We hypothesized that the participants’ functional health literacy levels were associated with various demographic characteristics. Therefore, we designed a four-section questionnaire including the following information: (1) age, gender, and education; (2) self-assessed disease knowledge (ie, knowledge of disease in general rather than of particular diseases); (3) 3 validated health literacy assessment tools (ie, the All Aspects of Health Literacy Scale [AAHLS] [21], the eHealth Literacy Scale [eHEALS] [22], and the 6-item General Health Numeracy Test [GHNT-6] [23]); and (4) health beliefs and self-confidence measured by the Multidimensional Health Locus of Control (MHLC) Scales Form B [24]. Informed by relevant studies [6-10,25-35], we hypothesized that the participants’ functional health literacy status could be closely associated with their health literacy status measured by the AAHLS, the eHEALS, and the GHNT-6, and their health beliefs evaluated by the MHLC Form B.

### Informant Recruitment and Web-Based Survey

Using randomized sampling, we recruited survey participants from Qilu Hospital affiliated to Shandong University, China. Those included in this study must (1) be 18 years or older, (2) have year 6 schooling or over to understand the questionnaire item, and (3) voluntarily participate in the survey. The questionnaire was administered via *wenjuanxing* [36], the most popular digital survey platform in China, from August 1, 2022, to August 31, 2022. A returned questionnaire was valid only when all question items included were answered, according to our predefined validation criterion.

### Data Collection, Coding, and Analysis

On September 1, 2022, the responses to the questionnaire were downloaded from *wenjuanxing*. We double-checked whether a response was provided to each question item to ascertain the validity of the data in each returned questionnaire. After that, all valid data were coded according to the predefined coding schemes of Likert scales with different point (score) ranges. Subsequently, we worked out essential statistical figures concerning the informants’ demographic information and calculated the sum scores of the following information: the subscales of the AAHLS, the 2 health literacy scales (the eHEALS and the GHNT-6), and the subscales of the MHLC. Finally, we used latent class analysis to classify Chinese populations into clusters of different functional health literacy (dependent variables) and identify significant factors closely associated with low functional health literacy (independent variables).

### Ethics Approval

This study was approved by the ethics review board of Qilu Hospital of Shandong University, China. The review number is KYLL-202208-026. Written consent was obtained from the patient participants. The data collected were anonymous or deidentified for privacy and confidentiality protection. We recruited patients who were willing to support our research without compensation.

## Results

### Descriptive Statistics of the Information Collected

Table 1 shows the descriptive statistics of the data collected. All data in the 800 returned questionnaires proved valid according to the predefined validation criterion. The participants were aged 42.56 (SD 11.47) years on average. Of them, 430 (54%) were female. The mean score for education was 3.26 (SD 1.46), showing that the participants’ average educational attainment was between year 12 and diploma. The mean score for their self-assessed disease knowledge (mean 2.41, SD 0.95) indicates that they assessed their disease knowledge as between “knowing a lot” and “knowing some.” The mean scores of the subconstructs in the AAHLS ranged from 2.05 (SD 0.74) to 2.12 (SD 0.745) for functional health literacy, 1.72 (SD 0.75) to 1.88 (SD 0.75) for communicative health literacy, and 1.56 (SD 0.73) to 2.02 (SD 0.50) for critical health literacy. These mean values imply that they basically “sometimes” needed help to read health-related information; the frequency that they knew how to effectively communicate with doctors and nurses was between “always” and “sometimes,” and the frequency that they were critical about health information was between “always” and “sometimes,” respectively. The mean scores for the 8 items on the eHEAL scale ranged from 2.81 (SD 1.88) to 2.99 (SD 1.18), implying that they were more likely to disagree or feel unsure that they had the skills and knowledge that enabled them to navigate electronic health platforms and find helpful health-related information. Each participant returned an average of 2.52 (SD 1.23) correct responses to the 6 numeracy question items on the GHNT scale. This means that a large share of participants answered the 6 questions wrongly. As with their scoring performance on the MHLC Scales Form B, they averagely scored a sum of 18.78 (SD 4.69), 16.54 (SD 4.51), and 17.89 (SD 4.24) on the “Internal,” “Chance,” and “Powerful Others” subscales, respectively. The determined response of “slightly disagree” for the “Internal” subscale indicates that they somehow did not believe in their internal drives to stay healthy. The determined response between “moderately disagree” and “slightly disagree” for the “Chance” subscale implies that they were generally less likely to attribute their health to a matter of luck. The determined response between “moderately disagree” and “slightly disagree” for the “Powerful Others” subscale means that they generally did not believe in the role of others in the maintenance of their health.

**Table 1:** Descriptive statistics (N=800).

	Participants
Age (years), mean (SD)	42.56 (11.47)
Gender (female), n (%)	430 (54)
Education, mean (SD)	3.26 (1.46)
Disease knowledge, mean (SD)	2.41 (0.95)
FHL1^a^, mean (SD)	2.05 (0.74)
FHL2^b^, mean (SD)	2.11 (0.96)
FHL3^c^, mean (SD)	2.12 (0.74)
COHL1^d^, mean (SD)	1.72 (0.75)
COHL2^e^, mean (SD)	1.87 (0.75)
COHL3^f^, mean (SD)	1.88 (0.75)
CRHL1^g^, mean (SD)	1.97 (0.76)
CRHL2^h^, mean (SD)	1.93 (0.72)
CRHL3^i^, mean (SD)	1.93 (0.75)
CRHL4^j^, mean (SD)	2.02 (0.73)
CRHL5^k^, mean (SD)	1.98 (0.73)
CRHL6^l^, mean (SD)	1.56 (0.50)
eHEAL1^m^, mean (SD)	2.81 (1.18)
eHEAL2^n^, mean (SD)	2.88 (1.19)
eHEAL3^o^, mean (SD)	2.87 (1.18)
eHEAL4^p^, mean (SD)	2.99 (1.18)
eHEAL5^q^, mean (SD)	2.85 (1.20)
eHEAL6^r^, mean (SD)	2.89 (1.21)
eHEAL7^s^, mean (SD)	2.93 (1.19)
eHEAL8^t^, mean (SD)	2.87 (1.19)
GHNT_SUM_Correct^u^, mean (SD)	2.52 (1.23)
Internal_Sum^v^, mean (SD)	18.78 (4.69)
Chance_Sum^w^, mean (SD)	16.54 (4.51)
PowerfulOthers_Sum^x^, mean (SD)	17.89 (4.24)
Valid N (listwise)	800

^a^Functional Health Literacy Item 1: How often do you need someone to help you when you are given information to read by your doctor, nurse, or pharmacist?

^b^Functional Health Literacy Item 2: When you need help, can you easily get hold of someone to assist you?

^c^Functional Health Literacy Item 3: Do you need help to fill in official documents?

^d^Communicative Health Literacy Item 1: When you talk to a doctor or nurse, do you give them all the information they need to help you?

^e^Communicative Health Literacy Item 2: When you talk to a doctor or nurse, do you ask the questions you need to ask?

^f^Communicative Health Literacy Item 3: When you talk to a doctor or nurse, do you make sure they explain anything that you do not understand?

^g^Critical Health Literacy Item 1: Are you someone who likes to find out lots of different information about your health?

^h^Critical Health Literacy Item 2: How often do you think carefully about whether health information makes sense in your particular situation?

^i^Critical Health Literacy Item 3: How often do you try to work out whether information about your health can be trusted?

^j^Critical Health Literacy Item 4: Are you the sort of person who might question your doctor or nurse’s advice based on your own research?

^k^Critical Health Literacy Item 5: Do you think that there are plenty of ways to have a say in what the government does about health?

^l^Critical Health Literacy Item 6: What do you think matters most for everyone’s health? (1) information and encouragement to lead healthy lifestyles; (2) good housing, education, decent jobs, and good local facilities.

^m^Electronic Health Literacy Item 1: I know what health resources are available on the internet.

^n^Electronic Health Literacy Item 2: I know where to find helpful health resources on the internet.

^o^Electronic Health Literacy Item 3: I know how to find helpful health resources on the internet.

^p^Electronic Health Literacy Item 4: I know how to use the internet to answer my health questions.

^q^Electronic Health Literacy Item 5: I know how to use the health information I find on the internet to help me.

^r^Electronic Health Literacy Item 6: I have the skills I need to evaluate the health resources I find on the internet.

^s^Electronic Health Literacy Item 7: I can tell high-quality from low-quality health resources on the internet.

^t^Electronic Health Literacy Item 8: I feel confident in using information from the internet to make health decisions.

^u^General Health Numeracy Test Item 1: Call your doctor if you have a temperature of 100.4 F or greater. The thermometer looks like the following: 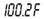. Do you call the doctor? General Health Numeracy Test Item 2: If 4 people out of 20 have a chance of getting a cold, what would be the risk of getting a cold? General Health Numeracy Test Item 3: Suppose that the maximum heart rate for a 60-year-old woman is 160 beats per minute and that she is told to exercise at 80% of her maximum heart rate. What is 80% of that woman’s maximum heart rate? General Health Numeracy Test Item 4: You ate half the container of carrots. How many grams of carbohydrates did you eat? General Health Numeracy Test Item 5: Your doctor tells you that you have high cholesterol. He informs you that you have a 10% risk of having a heart attack in the next 5 years. If you start on a cholesterol-lowering drug, you can reduce your risk by 30%. What is your 5-year risk if you take the drug? General Health Numeracy Test Item 6: A mammogram is used to screen women for breast cancer. False positives are tests that incorrectly show a positive result. A total of 85% of positive mammograms are actually false positives. If 1000 women receive mammograms and 200 are told that there is an abnormal finding, how many women are likely to actually have breast cancer?

^v^The Internal Locus of Control: Beliefs that one’s health is up to their own actions and behaviors.

^w^The Chance Locus of Control: Beliefs that one’s health is up to fate, chance, or luck.

^x^The Powerful Others Locus of Control: Beliefs that one’s health is up to others’ actions and behaviors.

### Latent Class Modeling

#### Model Fit Statistics

**Figure 1:**
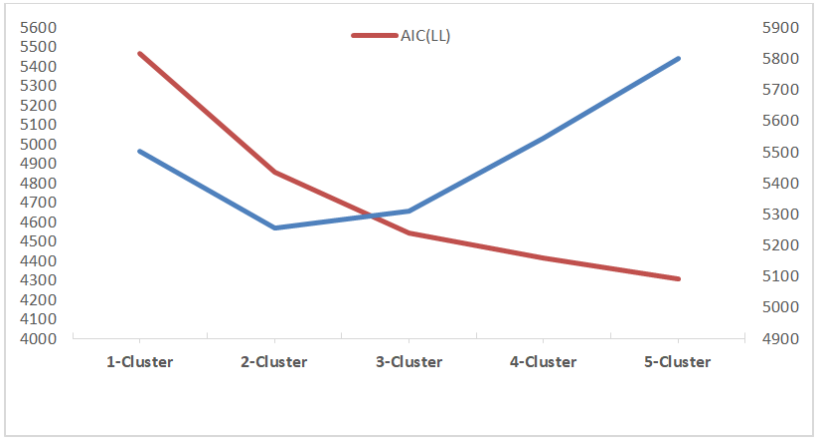
Changes in model fit statistics. AIC: Akaike information criterion; LL: log-likelihood.

Tables 2 and 3 and Figure 1 show the model fit statistics of the latent class analysis. The Akaike information criterion and the Bayesian information criterion provide measures of model performance. Smaller Akaike information criterion and Bayesian information criterion are indicative of better model performance. Indexes like the Lo-Mendell-Rubin likelihood ratio test and the bootstrap likelihood ratio test examined whether adding clusters would significantly improve model performance. We took all the factors into consideration and decided to opt for a 3-cluster solution for better model performance and simplicity to guide the subsequent qualitative analyses.

**Table 2:** Model fit statistics (1).

Models	LL^a^	BIC(LL)^b^	AIC(LL)^c^	AIC3(LL)^d^	Npar^e^	*L* ^2^ ^f^	*df*	*P* value^g^
1-Cluster	–2727.86	5502.51	5469.72	5476.72	7.00	5455.72	793.00	<.001
2-Cluster	–2343.64	5255.47	4857.28	4942.28	85.00	4687.28	715.00	<.001
3-Cluster	–2110.54	5310.67	4547.08	4710.08	163.00	4221.08	637.00	<.001
4-Cluster	–1966.50	5543.99	4415.00	4656.00	241.00	3933.00	559.00	<.001
5-Cluster	–1834.60	5801.60	4307.21	4626.21	319.00	3669.21	481.00	<.001

^a^LL: log-likelihood. The smaller the absolute value of LL, the better the model fit.

^b^BIC: Bayesian information criterion. Values closer to 0 indicate better fit.

^c^AIC: Akaike information criterion. Values closer to 0 indicate better fit.

^d^AIC3: Akaike information criterion 3.

^e^Npar: number of estimated parameters.

^f^*L^2^*, *df*: the sample size–adjusted BIC (SABIC) based on the *L*^2^ and *df*, which is the more common formulation in the analysis of frequency tables. They are defined as follows: BIC*_L_*^2^ = *L*^2^ *–* log(*N*)*df*, AIC*_L_*^2^ = *L*^2^ *–* 2*df*, AIC3*_L_*^2^ = *L*^2^ *–* 3*df*, CAIC*_L_*^2^ = *L*^2^ *–* (log(*N*)+1)*df*, SABIC*_L_*^2^ = *L*^2^ *–* log((*N*+2)/24)*df.* These information criteria weight the fit and the parsimony of a model: the lower the BIC, AIC, AIC3, CAIC, or SABIC, the better the model.

^g^All *P* values <.01.

**Table 3:** Model fit statistics (2).

Models	Bootstrap *P*^a^	VLMR^b^	*P* value	–2LL Diff^c^	Bootstrap *P*	Class Err^d^	Entropy *R*^2^^e^
1-Cluster	.54	N/A^f^	N/A	N/A	N/A	0.00	1.00
2-Cluster	.16	768.44	<.001	768.44	.00	0.00	0.97
3-Cluster	.04	466.20	<.001	466.20	.00	0.00	0.99
4-Cluster	.02	288.08	<.001	288.08	.00	0.00	0.99
5-Cluster	.00	263.79	<.001	263.79	.00	0.00	0.99

^a^Bootstrap *P*: if *P*<.05, the *k*-class model is selected over the *k* – 1 class model. Rather than relying on the asymptotic *P* value, it is also possible to estimate the *P* value associated with the goodness-of-fit ^2^ statistics by means of a parametric bootstrap.

^b^VLMR: Vuong-Lo-Mendell-Rubin test. It is used to test if a model with *k* classes is better than model with *k* *–* 1 class (eg, a 3-class vs a 2-class model). We recommend conducting and reporting VLMR tests where applicable.

^c^−2LL Diff: −2 log-likelihood difference.

^d^Class Err: class error.

^e^Entropy *R*^2^: values >0.8 indicate high degree of separation between classes.

^f^N/A: not applicable.

#### Latent Class Profiling

**Figure 2:**
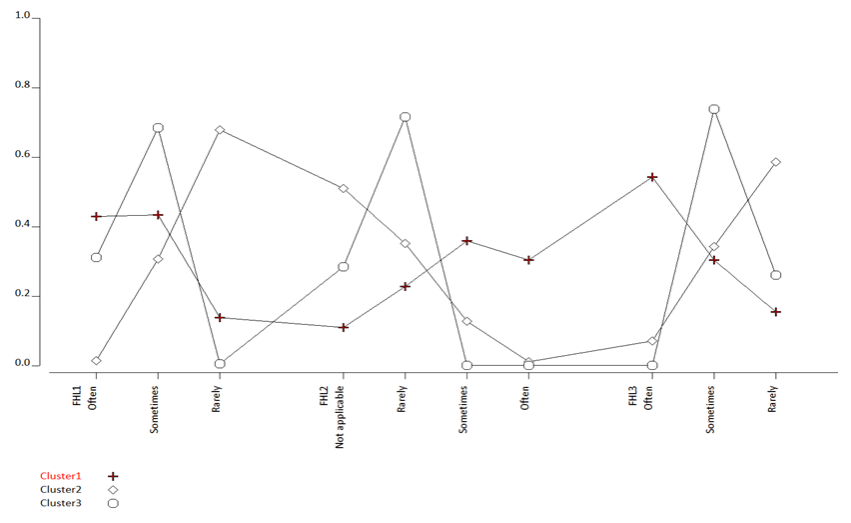
Conditional probabilities of responses to FHL items. FHL: Functional Health Literacy.

Figure 2 and Table 4 show the distribution of conditional probabilities of responses (often, sometimes, and rarely) to each of the 3 items of the Functional Health Literacy (FHL) scale within each latent cluster. Conditional probabilities of responses are mutually exclusive and sum to 1, based on the conditional independence assumption that underlines latent class analysis. For example, Figure 1 illustrates the average probability of each response given the cluster membership assigned to a study participant. Responses of the highest conditional probability were the most likely responses from study participants in a certain latent cluster. For example, we observed that participants assigned to cluster 1 preferred the first (“often”) (conditional probability [CP] 0.36) and second responses (“sometimes”) (CP 0.38) to the first item of the FHL scale (FHL1): “How often do you need someone to help you when you are given information to read by your doctor, nurse, or pharmacist?” Participants in cluster 1 also preferred the second (“rarely”) (CP 0.36) and third response (“sometimes”) (CP 0.33) to the second question of the FHL scale (FHL2): “When you need help, can you easily get hold of someone to assist you?” Lastly, participants in cluster 1 expressed that they “often” (CP 0.35) or “sometimes” (CP 0.40) needed help to fill in official documents (FHL3).

Participants in cluster 2 expressed that they “rarely” (CP 0.48) needed someone to help them when they were given information to read by their doctor, nurse, or pharmacist (FHL1). They also “rarely” (CP 0.51) needed others’ help to fill in official documents (FHL3). But interestingly, participants were less likely to think about seeking others’ help (not applicable, CP 0.64) or were “rarely” (CP 0.25) to easily get hold of someone to assist them when they needed help.

Participants in cluster 3 had “sometimes” as their preferred response to FHL1 (CP 0.89) and FHL3 (CP 0.67), suggesting that they had moderate functional health literacy to read, comprehend health information, and complete official documents by themselves. However, participants in cluster 3 expressed their concern regarding their ability to secure external help when in need, as they were “rarely” able to “easily get hold of someone to assist them when they needed help” (FHL3, CP 0.79).

On the basis of the observed response patterns across study participants, we thus labeled the 3 clusters as low functional health literacy (cluster 1), moderate-to-adequate functional health literacy (cluster 2), and low-to-moderate functional health literacy (cluster 3).

**Figure 3:**
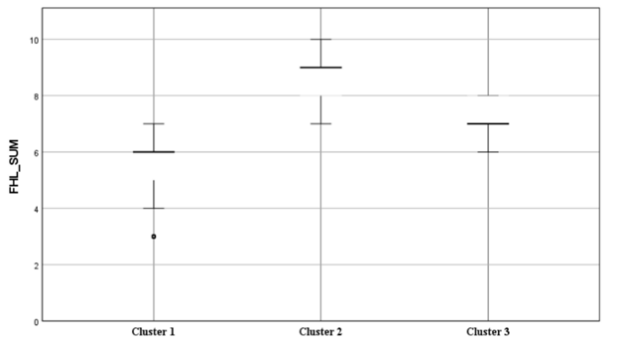
Boxplots of the sum of FHL of the latent classes. FHL: Functional Health Literacy.

Figure 3 and Table 5 show descriptive statistics of the 3 latent clusters representing the 3 levels of functional health literacy among the study participants. The low functional literacy group (cluster 1; n=292) had a mean of 5 (SD 1.01), which represented 36.5% of the total sample. The moderate-to-adequate functional health literacy group (cluster 2; n=286) had a mean of 8.5 (SD 0.93), representing 35.7% of the total sample size. The low-to-moderate functional health literacy group (cluster 3; n=222) had a mean of 7 (SD 0.56), representing the remaining 27.8% of the study population.

We compared the differences in pairwise cluster comparison. The result of the Games-Howell test in Table 6 shows that there were statistically significant differences among the 3 clusters representing the 3 levels of functional health literacy among the Chinese study sample.

**Table 4:** Conditional probabilities of responses within each latent cluster.

FHL^a^ item and response		Cluster 1	Cluster 2	Cluster 3
Cluster size		0.46	0.35	0.19
**FHL1**				
	Often	0.36	0.20	0.07
	Sometimes	0.38	0.33	0.89
	Rarely	0.26	0.48	0.04
	Total probability	1	1	1
**FHL2**				
	Not applicable	0.08	0.64	0.21
	Rarely	0.36	0.25	0.79
	Sometimes	0.33	0.08	0.00
	Often	0.23	0.03	0.00
	Total probability	1	1	1
**FHL3**				
	Often	0.35	0.13	0.10
	Sometimes	0.40	0.36	0.67
	Rarely	0.25	0.51	0.23
	Total probability	1	1	1

^a^FHL: Functional Health Literacy.

**Table 5:** Descriptive statistics of the latent clusters (N=800).

Clusters	Participants, n (%)	Mean	SD	SE
Cluster 1 (low FHL^a^)	292 (36.5)	5.46	1.01	0.06
Cluster 2 (moderate-to-adequate FHL)	286 (35.7)	8.55	0.93	0.06
Cluster 3 (low-to-moderate FHL)	222 (27.8)	7.24	0.56	0.04
Total	800	7.06	1.58	0.06

^a^FHL: functional health literacy.

**Table 6:** Multiple comparisons of intercluster differences.

(I) Cluster and (J) Cluster			Mean difference (I-J)		SE		*P* value		95% CI		
									Lower bound		Upper bound
**1**											
	2	–3.087^a^		0.08		<.001		–3.28		–2.90	
	3	–1.780^a^		0.07		<.001		–1.94		–1.62	
**2**											
	1	3.087^a^		0.08		<.001		2.90		3.28	
	3	1.307^a^		0.07		<.001		1.15		1.46	
**3**											
	1	1.780^a^		0.07		<.001		1.62		1.94	
	2	–1.307^a^		0.07		<.001		–1.46		–1.15	

^a^The mean difference is significant at the .05 level.

## Discussion

### Principal Findings in Relation to Previous Studies

We identified 3 latent clusters among the study participants with varying functional health literacy. People assigned to the first cluster had the lowest ability to read and comprehend health information and need others’ help to complete official documents but had a higher ability to identify and secure others’ help. People assigned to the second cluster had a relatively higher ability to read and comprehend health information and complete official documents independently, but they were not likely to consider seeking others’ help when they were in need. People assigned to the third cluster had a moderate-to-low ability to read and comprehend health information and to complete official documents independently but had the lowest ability to identify and secure others’ help. We thus labeled people in cluster 1 as having low functional health literacy and people in cluster 2 as having moderate-to-adequate functional health literacy noting that individuals were unlikely to seek external help when in need despite their higher ability to read and comprehend health information and complete official documents independently. Lastly, we described people assigned to the third cluster as having low-to-moderate functional health literacy, given their preferred responses of “sometimes” and “rarely” across the 3 items of the FHL scale. We identified some factors closely associated with low functional health literacy, as reported in the following principal findings.

#### Principal Finding 1: Males Were More Likely to Have Low Functional Health Literacy

Table 7 presents that when the study participants were men, they were most likely to belong to the low functional health literacy group (cluster 1 in Table 7). In contrast, female participants had the highest probability of falling into the moderate-to-adequate functional health literacy group (cluster 2 in Table 7). This gender difference has also been identified in previous studies that found that men were more likely to have lower health literacy skills than women [37-39]. This may be explained by the following facts: females became much more familiar with navigating the health care system during tackling health issues [38], they reported more health problems and had higher levels of medical service use and charges [40], and they played the traditional roles of taking care of sick family members and children [41].

**Table 7:** Posterior probabilities of gender across the latent clusters.

Gender	Cluster 1	Cluster 2	Cluster 3	Total probability
Male	0.40	0.33	0.27	1.00
Female	0.34	0.39	0.28	1.00

#### Principal Finding 2: People With Limited Education (Year 6-Year 12) Were Likely to Have Low Functional Health Literacy, and People With University Degrees Were More Likely to Identify and Secure Others’ Medical Help

According to Table 8, study participants with year 6 to year 12 education had the highest probability of falling into the low functional health literacy group (cluster 1 in Table 8). In contrast, participants with diploma education and postgraduate education were more likely to belong to the moderate-to-adequate functional health literacy group (cluster 2 in Table 8), and those with university education had the highest probabilities of having low-to-moderate functional health literacy group (cluster 3 in Table 8). This finding of our supports the findings reported in some previous studies that higher levels of health literacy were associated with higher levels of educational attainment, and lower levels of health literacy were associated with lower levels of educational attainment [38,39,42-44].

**Table 8:** Posterior probabilities of educational levels across the latent clusters.

Education	Cluster 1	Cluster 2	Cluster 3	Total probability
Year 6	0.46	0.36	0.17	1
Year 9	0.47	0.30	0.23	1
Year 12	0.36	0.35	0.29	1
Diploma	0.35	0.38	0.27	1
University	0.28	0.34	0.38	1
Postgraduate	0.07	0.61	0.32	1

#### Principal Finding 3: Low Functional Health Literacy Was Prevalent Among People Aged Between 38 and 68 years

Table 9 shows that when the study participants were aged between 38 and 68 years, they were more likely to be allocated into the low functional health literacy group (cluster 1 in Table 9). In contrast, those aged between 17 and 32 years had the highest probability of having low-to-moderate functional health literacy (cluster 3 in Table 9), and those aged between 33 and 37 years most probably belonged to the moderate-to-adequate functional health literacy group (cluster 3 in Table 9). This finding parallels some previous findings: health literacy skills were independently correlated with age, with growing age being significantly associated with declining health literacy skills [45-47]. The underlying reason may be the increasing cognitive dysfunction with the rise in age [47-49].

**Table 9:** Posterior probabilities of age groups across the latent clusters.

Age (years)	Cluster 1	Cluster 2	Cluster 3	Total probability
17-32	0.26	0.33	0.41	1
33-37	0.32	0.43	0.25	1
38-46	0.39	0.34	0.27	1
47-53	0.44	0.29	0.27	1
54-68	0.42	0.36	0.22	1

#### Principal Finding 4: People With Lower Functional Health Literacy Were More Likely to Be of Lower Self-efficacy

As shown in Table 10, when the study participants had lower functional health literacy, they were more likely to have lower levels of belief in the importance of self-discipline and internal drive to manage their health (cluster 1 in Table 10). In contrast, when the study participants had low-to-moderate functional health literacy, they were more likely to have higher self-efficacy (cluster 3 in Table 10), and when the study participants had moderate-to-adequate functional health literacy, they were more likely to have the highest levels of self-efficacy (cluster 2 in Table 10). There is no literature dealing with the link between self-efficacy and functional health literacy. This gap warrants relevant studies in the future.

**Table 10:** Posterior probabilities of Multidimensional Health Locus of Control (Form B) internal subscale sum across the latent clusters.

Internal_Sum	Cluster 1	Cluster 2	Cluster 3	Total probability
1-14	0.36	0.35	0.29	1
15-17	0.42	0.32	0.27	1
18-19	0.43	0.35	0.22	1
20-22	0.35	0.30	0.35	1
23-36	0.28	0.43	0.29	1

#### Principal Finding 5: Chinese People With Low Functional Health Literacy Were More Likely to Believe That Staying Healthy Was a Matter of Luck

It is clear from Table 11 that participants with low functional health literacy were more likely to be believers in maintaining their health as a matter of luck (cluster 1 in Table 11). In contrast, participants with low-to-moderate functional health literacy were less likely to believe that maintaining their health was a matter of luck (cluster 3 in Table 11), and participants with moderate-to-adequate functional health literacy were the least likely to believe the maintenance of their health as a matter of luck (cluster 2 in Table 11). No literature can be found to investigate the association between the belief that staying healthy is a matter of luck and low functional health literacy. This gap needs to be filled.

**Table 11:** Posterior probabilities of Multidimensional Health Locus of Control (Form B) chance subscale sum across the latent clusters.

Chance_Sum	Cluster 1	Cluster 2	Cluster 3	Total probability
6-12	0.19	0.48	0.32	1
13-15	0.35	0.28	0.37	1
16-17	0.41	0.35	0.24	1
18-19	0.39	0.29	0.32	1
20-36	0.47	0.31	0.22	1

### Implications

This study can provide some implications for health education, health research, and public health policy making. The 5 factors significantly associated with low functional health literacy can serve as important indicators for screening people with low functional health literacy to deliver more targeted education and more effective interventions. Knowledge, skills, beliefs, and practices related to the 6 associated factors could be integrated into public health education about functional health literacy. Researchers may gain some insights into the topic of low functional health literacy and associated factors. Finally, our research results and findings are likely to provide some implications for future public health policy making.

### Limitations

This study has some limitations. First, self-reported literacy skills and health beliefs and self-confidence are likely to incur some reporting bias. The self-assessed health literacy and health beliefs and self-confidence are not always consistent with the participant’s actual health literacy skills and real status of health beliefs and self-confidence. More objective measures need to be developed to increase the reliability and consistency of assessment of health literacy skills and health beliefs and self-confidence among culturally and linguistically diverse populations. Second, the generalizability of our research results and findings could be somehow limited. We recruited participants from only one hospital, possibly making our results and findings less generalizable to populations in other provinces in China and to populations in different sociocultural communities globally. Further studies need to be conducted to verify the results and findings of our study among populations of diverse ethnic and sociocultural backgrounds.

### Conclusions

We classified Chinese populations into low, low-to-moderate, and moderate-to-adequate functional health literacy groups and identified five factors associated with low communicative health literacy: (1) male gender (40-49 years), (2) lower educational attainment (below diploma), (3) age between 38 and 68 years, (4) lower self-efficacy, and (5) belief that staying healthy was a matter of luck. These associated factors can provide some implications for health education, medical research, and health policy making.

## Abbreviations

AAHLS: All Aspects of Health Literacy Scale
CP: conditional probability
eHEALS: eHealth Literacy Scale
FHL: Functional Health Literacy scale
GHNT-6: 6-item General Health Numeracy Test
MHLC: Multidimensional Health Locus of Control

## Data Availability

Data are available upon reasonable request via victorsyhz@hotmail.com.
